# The prevalence and genetic characteristics of porcine circovirus type 2 and 3 in Korea

**DOI:** 10.1186/s12917-018-1614-x

**Published:** 2018-09-26

**Authors:** Seung-Chai Kim, Salik Nazki, Sam Kwon, Jhung-Hyun Juhng, Kyeong-Hwan Mun, Do-Young Jeon, Chang-Gi Jeong, Amina Khatun, Seog-Jin Kang, Won-Il Kim

**Affiliations:** 1 College of Veterinary Medicine, Chonbuk National University, 79 Gobong-ro, Iksan, 54596 Jeonbuk Republic of Korea; 2 National Institute of Animal Science, Rural Development Administration, Cheonan, 55365 South Korea

**Keywords:** PCV2, PCV3, Prevalence, Genotyping, Genetic characteristics

## Abstract

**Background:**

Porcine circovirus-associated diseases (PCVAD), caused by porcine circovirus type 2 (PCV2), threaten the pig industry worldwide. Five genotypes of PCV2 were recently identified: PCV2a, PCV2b, PCV2c, PCV2d and PCV2e. In addition, a novel porcine circovirus from a case of a sow with dermatitis, nephropathy syndrome and reproductive failure has been identified based on metagenomic analysis and classified as porcine circovirus type 3 (PCV3). Therefore, the current study was conducted to determine the prevalence and genetic characteristics of PCV2 and PCV3 in clinical samples.

**Results:**

A total of 471 samples (161 tissue samples of lungs and lymph nodes from 34 farms and 310 serum samples from 47 farms) were tested for PCV2. Among them, 171 samples from 59 farms that had been positive for PCV2 were genotyped. Another 690 samples (296 tissue samples of lungs and lymph nodes from 91 farms, 108 samples of aborted foetuses from 26 farms, and 286 serum samples from 47 farms) were tested for PCV3. Based on PCV2 genotyping results, PCV2d was the most prevalent genotype (107 of 171 samples), and co-infections with combinations of PCV2a, 2b and 2d were identified in 48 samples from 17 farms. A total of 14 samples from 11 farms were also positive for both PCV2 and PCV3. For PCV3, 57 samples (9.8%) from 32 farms (23.2%) were positive. Among the 108 aborted foetuses from 26 farms, only 2 samples were positive for PCV3. Based on sequence comparisons, PCV2d shares 89.6–91.0% and 93.2–94.3% homology with PCV2a and PCV2b, respectively; 98.6–100% homology is shared among PCV2d strains. The PCV3 strains identified in this study share 98.0–99.5% homology.

**Conclusions:**

Our study concludes that PCV2d has become the most predominant genotype in Korea. PCV3 was also identified in clinical samples, though no significant association with clinical symptoms was observed in PCV3-positive cases.

## Background

Porcine circovirus (PCV), belonging to the family *Circoviridae* and the genus *Circovirus*, is the smallest virus with a circular, ambisense, single-stranded DNA genome. Two types of PCVs are known to be infectious to pigs: PCV1 and PCV2. PCV1 was first detected in 1974 [1]; non-pathogenic to pigs, PCV1 was found as a contaminant of PK-15 cell culture [2]. Reported as a new syndrome, post-weaning multi-systemic wasting syndrome (PMWS), PCV2 was first identified in 1996 [3, 4]. Unlike PCV1, PCV2 is one of the most important pathogens in the swine industry because of its association with numerous types of syndromes and diseases in pigs under the umbrella of porcine circovirus-associated disease (PCVAD) [5, 6], including reproductive failure [7], porcine respiratory disease complex (PRDC) [8], porcine dermatitis and nephropathy syndrome (PDNS) [9], granulomatous enteritis [10, 11], proliferative and necrotizing pneumonia (PNP) [12], necrotizing lymphadenitis, and possibly exudative epidermitis [13].

The capsid protein of PCV2, which is encoded by ORF2, binds to the host receptor and triggers immune responses [14, 15]. Recombination mainly occurs within ORF1, though it can occur in ORF2 [16]. As the range of nucleotide variation is greater for ORF2 than for ORF1 [17–19], ORF2 genome analysis is mainly used for phylogenetic and epidemiological markers and is considered similar to PCV2 whole-genome analysis [20, 21]. In 2008, the EU consortium on PCVAD set standardized nomenclature guidelines for PCV2 genotypes [22], indicating that the p-distance cut-off value should be greater than 0.035 [23, 24]. With this criterion, 5 genotypes of PCV2 have been identified: PCV2a, PCV2b, PCV2c, PCV2d and PCV2e. Until the early 2000s, PCV2a was the dominant PCV2 genotype worldwide, and it can be subdivided into four clusters (2A to 2D) [20, 21]. In contrast, PCV2b, which can be subdivided into three clusters (1A to 1C), was the dominant strain worldwide after 2000 [20, 21, 25]. PCV2c has only been detected in archived swine serum samples from Denmark [25]. PCV2d was first identified in China in 2009 [26]. After worldwide debate differentiating mPCV2b and PCV2d, PCV2d was finally proposed as an independent genotype that includes the mPCV2b cluster [21, 23, 27, 28]. PCV2d is currently the most dominant genotype [21] and is a source of much concern to the swine industry, which has reported worldwide PCV2 vaccine failure [29–32]. In 2016, PCV2e was identified in the US through diagnostic phylogenetics [33].

Recently, a novel porcine circovirus was identified through metagenomic analysis from a sow in the US suffering from dermatitis, nephropathy syndrome and reproductive failure; this isolate was classified as porcine circovirus type 3 (PCV3) [34]. PCV3 has also been detected in pigs with cardiac and multisystemic inflammation in the US [35]. Furthermore, PCV3 is being reported in China [36, 37] and Korea [38] as a new threat to the swine industry. However, insufficient evidence indicates that PCV3 will be as significant a pathogen as PCV2 because PCV3 was detected without any significant clinical symptoms [39]. As little information exists on overall porcine circovirus infection in Korea, this study was conducted to determine the prevalence and genetic characteristics of PCV2 and PCV3 in Korea.

## Methods

### Sample information

During a period of 1 year between April 2015 and March 2016, 471 samples (161 tissue samples of lungs and lymph nodes from 34 farms and 310 serum samples from 47 farms) were submitted to Chonbuk National University-Veterinary Diagnostic Center (CBNU-VDC) for PCV2 testing. Among the samples, 30 serum samples from 25 farms and 141 tissue samples from 34 farms were positive for PCV2 and genotyped. In addition, another set of 690 samples (296 tissue samples of lungs and lymph nodes from 91 farms, 108 samples of aborted foetuses from 26 farms, and 286 serum samples from 47 farms) submitted to CBNU-VDC between April 2016 and June 2017 were tested for PCV3.

### Nucleic acid extraction and PCR detection of PCV2

Tissue samples were homogenized, mixed with phosphate-buffered saline (PBS; 0.1 M, pH 7.4) and centrifuged at 2500 rpm for 10 min at 4 °C. Viral nucleic acid was immediately extracted from the supernatant using Patho Gene-spin DNA/RNA Extraction Kit (iNtRON Biotechnology Inc., Seongnam, Korea) according to the manufacturer’s instructions. Four sets of primers were used. One set of primer was used for detection and ORF2 genome sequencing [40], and three were used for subtype differential PCR [14, 41]. Two microlitres of extracted DNA and 2× F-Star Taq PCR Master Mix (BIOFACT Co., Daejeon, Korea) were mixed with 10 pmol of each subtype-specific primer (Table 1). The PCR conditions were as follows: pre-denaturation at 94 °C for 1 min, 35 cycles of denaturation at 94 °C for 30 s, annealing at 60 °C for 30 s, and extension at 72 °C for 1 min, and a final extension at 72 °C for 10 min.

**Table 1:** Primers used in this study

Primer name	Nucleotide sequence (5′ - 3′)	Product size	Purpose	Reference
F-PCV2C	GCT GGC TGA ACT TTT GAA AGT	1767 bp	PCV2 detection and sequencing	L. Li et al., 2016
R-PCV2C	AAA TTT CTG ACA AAC GTT ACA			
PCV2ab 2NF	GGT TGG AAG TAA TCA ATA GTG GA	277 bp	PCV2a-specific	T. Kwon et al., 2017
PCV2a 2NR	GGG GAA CCA ACA AAA TCT C			Hesse et al., 2008
PCV2ab 2NF	GGT TGG AAG TAA TCA ATA GTG GA	277 bp	PCV2b-specific	T. Kwon et al., 2017
PCV2b 2NR	GGG GCT CAA ACC CCC GCT C			Hesse et al., 2008
PCV2d 2NF	GGT TGG AAG TAA TCG ATT GTC CT	343 bp	PCV2d-specific	T. Kwon et al., 2017
PCV2d 2NR	TCA GAA CGC CCT CCT GGA AT			
PCV3–1-F	TTA CTT AGA GAA CGG ACT TGT AAC G	649 bp	PCV3 detection	Ku et al., 2016
PCV3–1-R	AAA TGA GAC ACA GAG CTA TAT TCA G			
PCV3-genome-2-F	TTG CAC TTG TGT ACA ATT ATT GCG	1075 bp	PCV3 sequencing	Ku et al., 2016
PCV3-genome-2-R	ATC TTC AGG ACA CTC GTA GCA CCA C			

### PCR detection of PCV3

Samples suspected of harbouring PCV3 had already been tested for porcine reproductive and respiratory syndrome (PRRS) and PCV2, as they are routinely diagnosed pathogens of pigs in the CBNU-VDC. Using 2× F-Star Taq PCR Master Mix (BIOFACT Co., Daejeon, Korea), a pair of primers was used to detect PCV3, and another pair of primers was utilized for ORF2-region genome sequencing, as described in a previous study [36] (Table 1). The following conditions were used for PCR: pre-denaturation at 94 °C for 2 min, 35 cycles of denaturation at 94 °C for 30 s, annealing at 56 °C for 30 s, and extension at 72 °C for 1 min, and a final extension at 72 °C for 10 min.

### Phylogenetic analysis

The ORF2 regions of PCV2 and PCV3 were amplified with the sequencing primers listed in Table 1. The PCR products were then sequenced using a commercial sequencing service (BIOFACT Co., Daejeon, Korea), and assembly was completed with SeqMan v5.06 (DNASTAR, Madison, Wisconsin, USA). A total of 32 sequences were submitted to GenBank, 27 of which were PCV2 and 5 PCV3 (Accession numbers MF631803-MF631834). A phylogenetic tree for PCV2 genotypes was inferred by using the maximum-likelihood (ML) method with 1000 replicates for bootstrap values based on the ORF2 scale and utilizing the software MEGA 6.06; Korean strains detected since 2015 and reference strains for each genotype were employed [42]. In the case of PCV3, the same method was used to reconstruct a phylogenetic tree on the ORF2 scale with all PCV3 ORF2 sequences in GenBank.

## Results

### PCR detection of PCV2 and genotyping

**Fig. 1:**
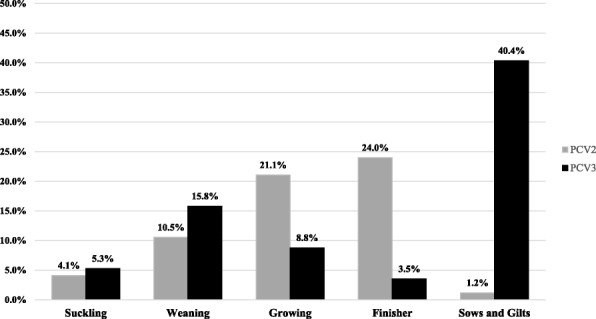
Distribution of PCV2 and PCV3 infection by age group

PCV2-positive samples were examined by genotyping PCR (Table 2). Serum samples were positive for PCV2a (1/30, 1 farm), PCV2b (1/30, 1 farm), PCV2d (25/30, 20 farms), and co-infection with PCV2b and 2d (3/30, 3 farms). Tissue samples were positive for PCV2a (8/141, 1 farm), PCV2b (5/141, 1 farm), PCV2d (82/141 17 farms), PCV2a and 2b (1/141, 1 farm), PCV2a and 2d (20/141, 3 farms), PCV2b and 2d (16/141, 8 farms), and PCV2a, 2b and 2d (9/141, 3 farms). In addition, 14 samples from 11 farms were positive for both PCV2 and PCV3 (Table 2). The age-wise distribution of PCV2-positive samples revealed that among five groups, finisher pigs were infected most (24.0%) prevalently, followed by growing pigs (21.1%), weaning pigs (10.5%), suckling pigs (4.1%) and sows and gilts (1.2%) (Fig. 1).

**Table 2:** PCR results for genotyping of PCV2-positive serum and tissue samples

Sample type	PCV2 genotype	Number of samples		Number of farms		PCV3 co-infection (samples/farms)
Serum Samples	2a	1	(1/30, 3.3%)	1	(1/25, 4%)	1 (1/1, 100%) /1 (1/1, 100%)
	2b	1	(1/30, 3.3%)	1	(1/25, 4%)	0
	2d	25	(25/30, 83.3%)	20	(20/25, 80%)	4 (4/25, 16%) /4 (4/20, 20%)
	2b, 2d	3	(3/30, 10%)	3	(3/25, 12%)	1 (1/3, 33.3%) /1 (1/3, 33%)
Tissue Samples	2a	8	(8/141, 5%)	1	(1/34, 2.9%)	0
	2b	5	(5/141, 3.1%)	1	(1/34, 2.9%)	0
	2d	82	(82/141, 51.3%)	17	(17/34, 50%)	6 (6/82, 7.3%) /3 (3/17, 17.6%)
	2a, 2b	1	(1/141, 0.6%)	1	(1/34, 2.9%)	1 (1/1, 100%) /1 (1/1, 100%)
	2a, 2d	20	(20/141, 12.5%)	3	(3/34, 8.8%)	1 (1/20, 5%) /1 (1/3, 33.3%)
	2b, 2d	16	(16/141, 10%)	8	(8/34, 23.5%)	0
	2a, 2b, 2d	9	(9/141, 5.6%)	3	(3/34, 8.8%)	0

### Phylogenetic analysis of PCV2 ORF2

**Fig. 2:**
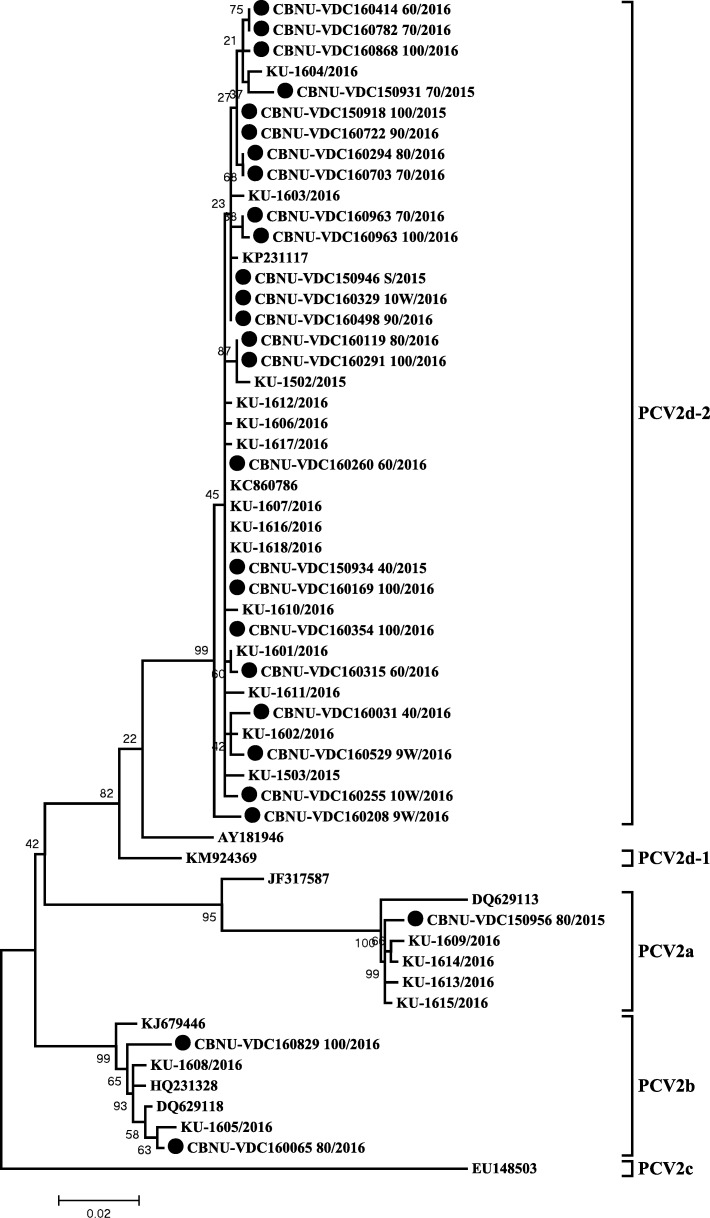
Phylogenetic trees of PCV2 ORF2 sequences constructed using the maximum-likelihood method based on the generalized time-reversible (GTR) model with G + I in MEGA 6.06. Bootstrap values were calculated with 1000 replicates. Filled circles indicate the PCV2 strains identified in this study

The 27 PCV2 ORF2 sequences identified in the current study were classified into three groups (PCV2a, PCV2b, and PCV2d), with minor mutant groups. All of the PCV2d sequences identified in this study belong to the PCV2d-2 clade (Fig. 2).

The ORF2 length of PCV2a and PCV2b is 702 bp, while that of PCV2d is 705 bp. Korean PCV2a, PCV2b, and PCV2d exhibit 99.4 ± 0.2% (99.1–99.6%), 98.7 ± 0.5% (98.1–99.4%), and 99.5 ± 0.3% (98.6–100%) within-group homology, respectively. In addition, PCV2d shares 90.4 ± 0.2% (89.6–91.0%) and 93.9 ± 0.3% (93.2–94.3%) homology with PCV2a and PCV2b, respectively, and PCV2a and PCV2b share 91.4 ± 0.3% homology (91.0–92.0%) (Table 3).

**Table 3:** Average nucleotide sequence homology within and between PCV2 genotypes

Nucleotide homology within genotypes (%)		Nucleotide homology between PCV2 genotypes (%)		
	Genotype	PCV2a	PCV2b	PCV2d
99.4 ± 0.2 (99.1–99.6)	PCV2a	–	–	–
98.7 ± 0.5 (98.1–99.4)	PCV2b	91.4 ± 0.3 (91.0–92.0)	–	–
99.5 ± 0.3 (98.6–100)	PCV2d	90.4 ± 0.2 (89.6–91.0)	93.9 ± 0.3 (93.2–94.3)	–

Homology was determined by the maximum-likelihood method based on the GTR model with G + I in MEGA 6.06 software. Bootstrap values were calculated with 1000 replicates. The analysis includes Korean ORF2 sequences isolated after 2015

### PCR detection of PCV3 and co-infection with PRRS virus (PRRSV) and PCV2

Among 286 serum samples from 47 farms, 37 samples (12.9%) from 20 farms (42.6%) were PCV3 positive. Of 296 tissue samples from 91 farms, 20 samples (6.8%) from 12 farms (6.8%) were positive. The positive rate of PCV3 was higher for serum samples than for tissue samples. Of 108 samples (26 farms) of aborted foetuses, only 2 samples (2 farms) were positive. The age-wise distribution of PCV3-positive samples revealed that among five groups, sows and gilts were infected most (40.4%) prevalently, followed by weaned pigs (15.8%), growing pigs (8.8%), suckling pigs (5.3%) and finisher pigs (3.5%) (Fig. 2). In addition, co-infection with PRRSV or PCV2 was identified in 12 serum and 13 tissue samples and in 4 serum and 7 tissue samples, respectively (Table 4).

**Table 4:** PCR results for PCV3-positive serum and tissue samples

Sample type	Number of samples	Number of farms	PRRS co-infection (samples/farms)	PCV2 co-infection (samples/farms)
Serum	37 (37/286, 12.9%)	20 (20/47, 42.6%)	12 (12/37, 32.4%)/10 (10/20, 50%)	4 (4/37, 10.8%)/4 (4/20, 20%)
Tissue	20 (20/296, 6.8%)	12 (12/91, 13.2%)	13 (13/20, 65%)/10 (10/12, 83.3%)	7 (7/20, 35%)/6 (6/12, 50%)
Aborted foetus	2 (2/108, 1.9%)	2 (2/26, 7.7%)	–	–

### Phylogenetic analysis of PCV3 ORF2

**Fig. 3:**
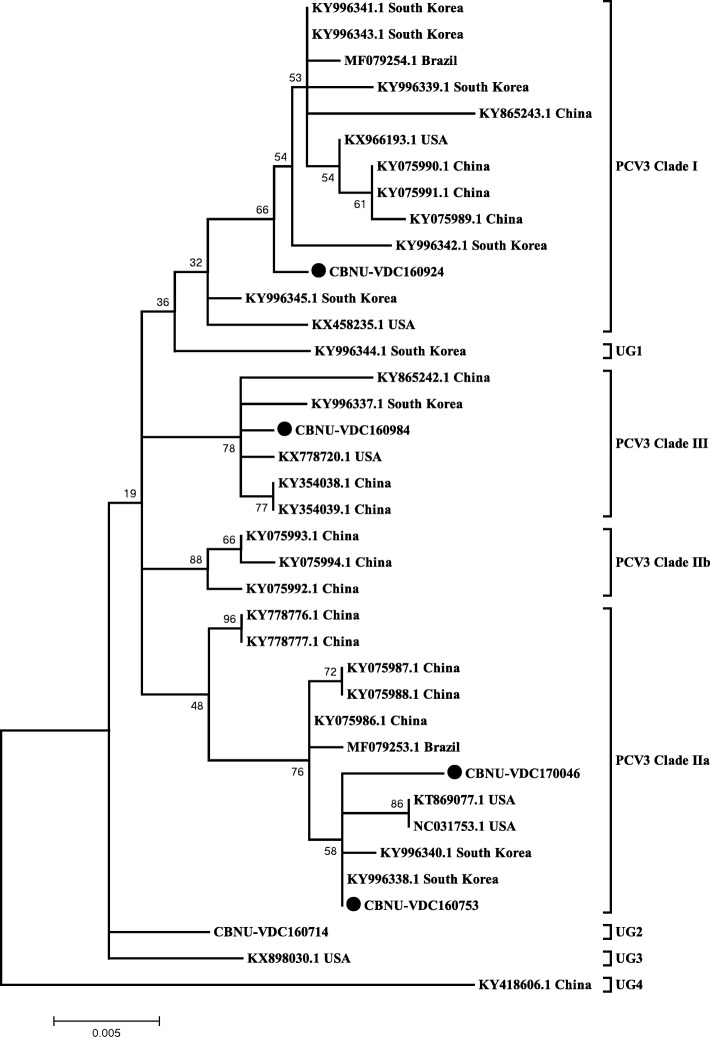
Phylogenetic trees of PCV3 ORF2 sequences of Korean PCV3 constructed using the maximum-likelihood method based on the GTR model with G + I in MEGA 6.06. Bootstrap values were calculated with 1000 replicates. Filled circles indicate the PCV3 strains identified in this study

The length of all PCV3 ORF2 sequences is 645 bp. Five PCV3 ORF2 sequences identified in this study were compared with those available in GenBank, and 98.7 ± 0.4% (98.0–99.5%) sequence homology is shared among the sequences. However, attempts at full-length sequencing were not successful. By comparing *p*-distances of all PCV3 ORF2 sequences present in GenBank, 3 clades, designated Clades I, II, and III, are proposed. Clade II can also be sub-grouped as Clade IIa and IIb. The Korean PCV3 strains are grouped into all three major clades (Fig. 3). Average *p*-distances of 0.0065 ± 0.0035, 0.0079 ± 0.0045, and 0.0050 ± 0.0026 were calculated for PCV3 Clades I, II, and III, respectively, and the *p*-distances between clades (Clade I/II: 0.0144 ± 0.0027, Clade I/III: 0.0152 ± 0.0027, and Clade II/III: 0.0155 ± 0.0030) were significant. However, four strains could not be classified into any of these three clades due to their long *p*-distances, suggesting the possibility of new clades or intermediate clades (Table 5).

**Table 5:** Average *p*-distances within and between PCV3 clades

Average *p*-distance within clades (mean ± SE)		Average *p*-distance between PCV3 clades (mean ± SE)						
	Genotype	Clade I	Clade II	Clade III	UG1	UG2	UG3	UG4
0.0065 ± 0.0035	Clade I	–	–	–	–	–	–	**–**
0.0079 ± 0.0045	Clade II	0.0144 ± 0.0027	–	–	–	–	–	**–**
0.0050 ± 0.0026	Clade III	0.0152 ± 0.0027	0.0155 ± 0.0030	–	–	–	–	**–**
–	UG1	0.0136 ± 0.0025	0.0163 ± 0.0031	0.0150 ± 0.0019	–	–	–	**–**
–	UG2	0.0132 ± 0.0022	0.0127 ± 0.0020	0.0134 ± 0.0019	0.0140	–	–	**–**
–	UG3	0.0148 ± 0.0022	0.0143 ± 0.0020	0.0150 ± 0.0019	0.0155	0.0109	–	**–**
–	UG4	0.0308 ± 0.0024	0.0293 ± 0.0017	0.0328 ± 0.0012	0.0341	0.0295	0.0279	**–**

The *p*-distance was determined by the neighbour-joining method based on the GTR model with G + I in MEGA 6.06 software. Bootstrap values were calculated with 1000 replicates. The analysis includes ORF2 sequences of 13 PCV3 Clade I, 15 PCV3 Clade II, 6 PCV3 Clade III and 4 ungrouped (UG) sequences (UG1 = KY996344.1, UG2 = CBNU-VDC160714, UG3 = KX898030.1 and UG4 = KY418606.1)

## Discussion

In the current study, PCV2 isolates detected in samples from 50 farms of 60 PCV2-positive farms were identified as PCV2d (Table 2 and Fig. 2), suggesting a major genotype shift from PCV2b to PCV2d on swine farms in Korea [14]. Detection of PCV2d alone was predominant in both serum and tissue samples, but co-detection of PCV2a, PCV2b or PCV2d was more prevalent in tissue samples such as lungs and lymph nodes than in serum samples. Since it is not possible to differentiate between PCV2 detected in tissue samples with current or previous infection and only actively replicating virus can be detected in circulating blood, PCV2 genotyping in serum samples was concluded to appropriately identify the major genotype causing clinical problems on farms at the specific time of the current study.

When the age-wise distribution of PCV2-positive cases was analysed, the highest positive rate was detected in finishers (24.0%), followed by growing pigs (21.1%). PCV2-positive rates were also reported in previous studies to be higher in growing and finisher pigs than in other groups, suggesting the higher susceptibility of growing and finisher pigs to PCV2 infection and maternal antibodies interfering with vaccination [43–45]. In addition, the PCV2-positive rate has increased in suckling and weaned pig populations (Fig. 1). Opriessnig et al. [46] reported that the current PCV2 vaccines, which were developed based on PCV2a, can prevent PCV2d transmission to naive pigs and the vaccinated pigs showed reduced levels of PCV2d viraemia. Because mass vaccination with PCV2a-based inactivated vaccines are applied to 3–4-week-old piglets and sows in Korean farms, it was concluded that the current PCV2 vaccines reduce clinical symptoms and lesions caused by PCV2d but do not provide sterile immunity against PCV2d infection.

Recently, clinical signs similar to those of PCVAD were reported from PCV2 negative pigs, and a novel porcine circovirus, PCV3, was identified from these cases [34]. Another research group reported a case of cardiac and multi-systemic inflammation due to PCV3 infection [35]. A Chinese research group detected PCV3 in China (34.7% of individual samples and 68.6% at the farm level were positive), suggesting the wide distribution of PCV3 in multiple tissues and the possibility of vertical transmission [36]. Nationwide surveillance for PCV3 has been conducted in Korea, reporting approximately 72.6% of investigated farms to be positive for this infection [38]. In the present study, serum and tissue samples from various farms were tested for PCV3, and 12.9% of serum samples (42.6% at the farm level) and 6.8% of tissue samples (13.2% at the farm level) were positive. Although the overall positive rate was less than that of the previous study by another Korean research group [38], PCV3 is clearly prevalent in the Korean swine industry.

Analysis based on age-wise distribution of PCV3-positive samples revealed the highest prevalence in the group of sows and gilts (40.4%), whereas the suckling pig group showed a relatively low positive rate (5.3%) (Fig. 1). In previous studies, PCV3 has been identified from sows that experienced abortion, and cardiac and multi-systemic inflammation [34–36]. Thus, the age susceptibility and physio-pathologic characteristic of PCV3 might have a close relationship with the high positive rate in the sow and gilt group. Reproductive failures and stillbirth cases have been reported to be caused by PCV3 [34, 36, 39]. In our study, however, PCV3 was detected from only 2 aborted foetuses from 2 farms (of 108 samples from 26 farms). In fact, the association of PCV3 with the clinical condition remains controversial. For example, Franzo G et al. [47] was unable to find statistically significant relevance of PCV3 co-infection with any clinical condition, yet Zhai et al. [48] reported that a higher genome load of PCV3 was detected in cases of severe respiratory disease or diarrhea than in mild cases. Thus, the relationship between PCV3 infection and clinical symptoms should be investigated further.

Genotyping PCV2 has been an important issue because different genotypes of PCV2 have resulted in outbreaks and vaccine failures in the global swine industry. PCV2 genotypes are divided by a *p*-distance cut-off of 0.035 [22]. PCV3 being one of the possible causes of PCVAD outbreak, it is indeed important to set criteria for genotyping of PCV3 and monitoring trends in genotype distribution. In a previous study, PCV3 strains were divided into 2 clades (PCV3a and PCV3b) based on the partial capsid protein gene of 474 bp [36]. In the present study, all PCV3 sequences available in GenBank were analysed based on the ORF2 sequence, and PCV3 was tentatively divided into three clades (Clades I, II and III). To define the *p*-distance cut-off for genotyping PCV3, more PCV3 ORF2 sequences should be identified worldwide.

## Conclusion

Our study summarized the recent evolution of PCV2 and PCV3 in Korea. PCV2d is most prevalent in Korea, even though PCV2a-based vaccines are currently used for regular vaccination. In addition, a novel porcine circovirus, PCV3, has recently emerged in Korea. Although no significant association was demonstrated between PCV3 infection and clinical symptoms, continuous surveillance for PCV3 should be performed in future studies.

## Abbreviations

CBNU-VDC: Chonbuk National University-Veterinary Diagnostic Center
PCV2: Porcine circovirus type 2
PCV3: Porcine circovirus type 3
PCVAD: Porcine circovirus-associated disease
*p*-distance: Pairwise distance
